# Immunotherapeutic effects of recombinant colorectal cancer antigen produced in tomato fruits

**DOI:** 10.1038/s41598-022-13839-1

**Published:** 2022-06-13

**Authors:** Se Hee Park, Kon-Young Ji, Seo Young Park, Hyun Min Kim, Sang Hoon Ma, Ju Hui Do, Hyuno Kang, Hyung Sik Kang, Doo-Byoung Oh, Jae Sung Shim, Young Hee Joung

**Affiliations:** 1 School of Biological Sciences and Technology, Chonnam National University, Gwangju, 61186 Korea; 2 Herbal Medicine Research Division, Korea Institute of Oriental Medicine, Daejeon, 34054 Korea; 3 Division of Analytical Science, Korea Basic Science Institute (KBSI), Daejeon, 34133 Republic of Korea; 4 Environmental Disease Research Center, Korea Research Institute of Bioscience and Biotechnology (KRIBB), Daejeon, 34141 Korea; 5 Department of Biosystems and Bioengineering, KRIBB School of Biotechnology, University of Science and Technology (UST), Daejeon, 34113 Korea

**Keywords:** Biotechnology, Plant sciences

## Abstract

The production of pharmacological vaccines in plants has been an important goal in the field of plant biotechnology. GA733-2, the protein that is also known as colorectal carcinoma (CRC)-associated antigen, is a strong candidate to produce a colorectal cancer vaccine. Tomato is the one of the major targets for production of an edible vaccine, as tomato is a fruit consumed in fresh form. It also contains high content of vitamins that aid activation of immune response. In order to develop an edible colorectal cancer vaccine, the transgene rGA733-Fc that encodes a fusion protein of GA733-2, the fragment crystallizable (Fc) domain, and the ER retention motif (rGA733-Fc) was introduced into tomato plants (*Solanum* *lycopersicum* cv. Micro-Tom). The transgenic plants producing rGA733-Fc (*rGA733-Fc*^*OX*^) protein were screened based on stable integration of transgene expression cassette and expression level of rGA733-Fc protein. Further glycosylation pattern analysis revealed that plant derived rGA733-Fc protein contains an oligomannose glycan structure, which is a typical glycosylation pattern found on ER-processing proteins. The red fruits of *rGA733-Fc*^*OX*^ transgenic tomato plants containing approximately 270 ng/g FW of rGA733-Fc protein were orally administered to C57BL/6 mice. Oral administration of tomato fruits of the rGA733-Fc expressing transgenic plants delayed colorectal cancer growth and stimulated immune responses compared to oral administration of tomato fruits of the h-Fc expressing transgenic plants in the C57BL/6J mice. This is the first study showing the possibility of producing an edible colorectal cancer vaccine using tomato plants. This research would be helpful for development of plant-derived cancer edible vaccines.

## Introduction

The recent vigorous isolation and characterization of tumor antigens has opened up the possibility for development of cancer vaccines. In the case of metastatic cancer, the therapeutic effect of immunotherapy with cancer vaccines is expected to be more effective than radiation treatment and chemotherapy^1–6^. The immune responses mediated by T cells and B cells lead to the removal of cancer cells, without affecting the surrounding normal cells in humans and animals^5–7^. In addition, the consistent maintenance of the immune response decreases the rate of cancer recurrence^8^. These findings provide strong evidences that cancer can be successfully treated with a recombinant antigen vaccine either alone or in conjunction with hormone therapy, chemotherapy or monoclonal antibody therapy^9–15^.

As a tool for pharmacological recombinant protein production, plants have several advantages compared with other production system, such as low production cost, appropriate post-translation modification, and lack of human pathogen contamination^16–23^. Another outstanding advantage of plant system for vaccine development is that plants producing pharmacological recombinant proteins can be used as edible vaccines^17^. Among the plants, tobacco system has been extensively used for production of vaccine candidates in plants. Despite of several benefits of tobacco system, such as easy transformation, robust expression of recombinant protein^24,25^, tobacco system is not the best target for development of edible vaccine. Thus, production of vaccine candidates in the edible plants will be an alternative for development of edible vaccines. Tomato is the most producing fruits worldwide due to its higher nutritional value and various uses as food ingredients^26^. In addition, tomato plants contain a high content of vitamins that may boost immune response^27^. There have been several cases that utilize tomato plants for pharmacological recombinant protein production^28–30^. For example, recombinant Norwalk virus (rNV) capsid protein was produced in tomato plants. The tomato-derived rNV capsid protein induced rNV specific serum IgG and mucosal IgA production in mice^30^. In addition, Hepatitis B virus (HBV) and Human papillomavirus (HPV) antigens was produced in tomato plants^28,29^.

GA733-2 is known to be a colorectal carcinoma antigen and an epithelial cell adhesion molecule (synonyms: EpCAM, TACSTD1, MIC18 and M4S1). The GA733-2 molecule is a 40-kDa transmembrane glycoprotein, and its molecular functions have been studied for three decades^31–37^. GA733-2 is not expressed in adult cells, but it is over-presented in human colorectal cancer and cancer-propagating cells^38^. Based on the specific expression pattern and its significant impact on induction of humoral immune response and suppression of colorectal cancer growth, GA733-2 has been sought to be a vaccine candidate for colorectal cancer^16,37,39^. However, low production rate of GA733-2 in plants is regarded as a limitation for development of edible colorectal plant vaccine. Several approaches have been devised to increase GA733-2 production in plants^40,41^. Among them, fusion of GA733-2 with fragment crystallizable (Fc) domain and ER retention peptide (rGA733-Fc) greatly increased GA733-2 production rate^40,41^. In addition, the rGA733-Fc recombinant protein successfully induced apoptosis and reduced tumorigenicity of MC38 and HCT116 carcinomal cells^41^. In this study, we attempted to generate transgenic tomato plant that expresses rGA733-Fc in its fruits, and determine its effects on colorectal cancer development using mice system. Western blot and ELISA analysis revealed that transgenic tomato plants produced up to 278.76 ng/g of rGA733-Fc recombinant protein in their fruits at red ripening stage. The plant-derived rGA733-Fc protein contained oligomannose glycan structure, which is a typical glycosylation pattern observed from ER-processed protein. Oral administration of red-ripened rGA733-Fc expressing tomato fruits delayed early growth of colorectal tumor and induced IgA production in mice.

## Results

### Stable expression of rGA733-Fc and human Fc in transgenic tomato plants

**Figure 1:**
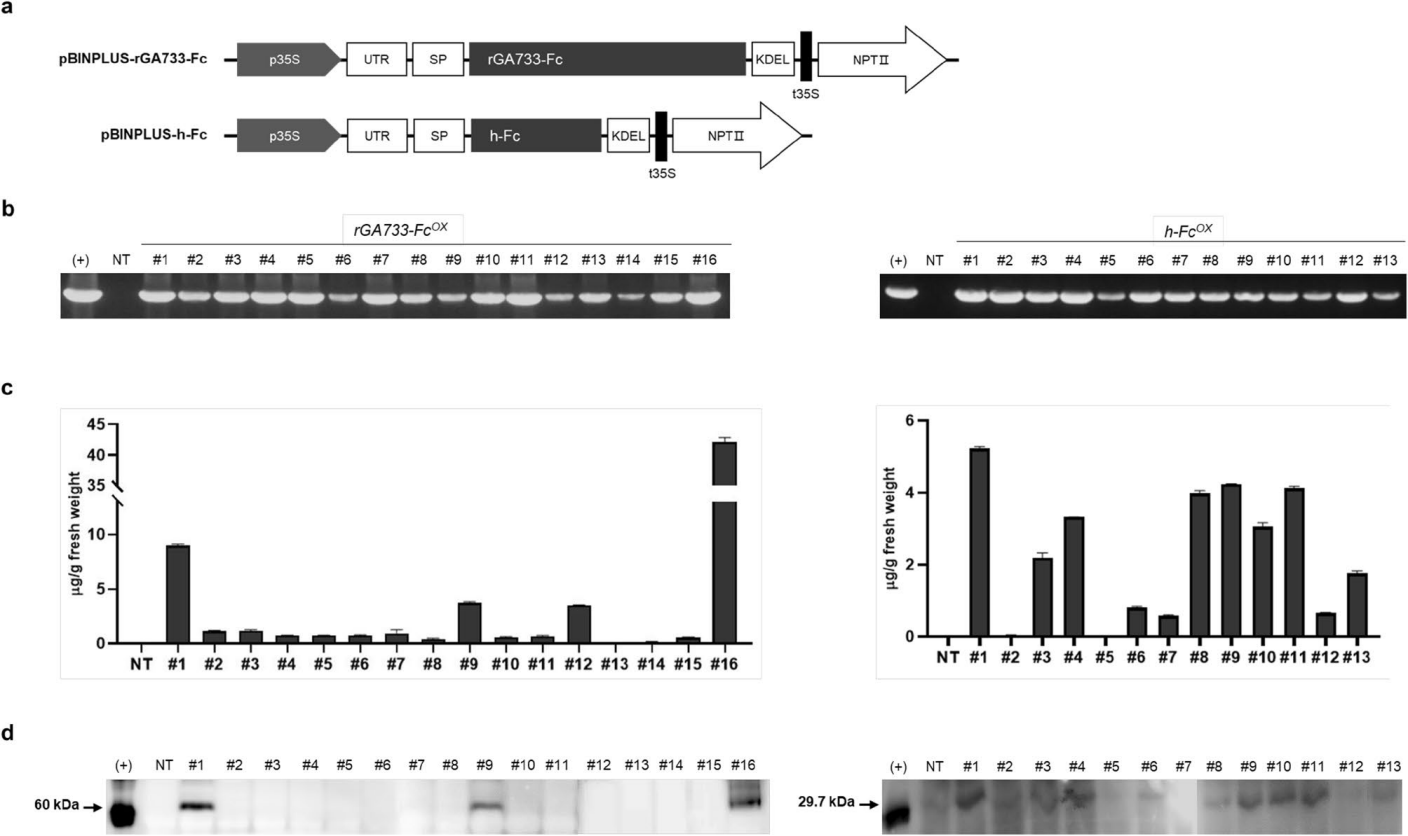
Production of rGA733-Fc and h-Fc expressing transgenic tomato. Transgenic tomato plants producing rGA733-Fc or h-Fc protein were produced using the Agrobacterium-mediated transformation method with pBINPLUS-GA733-Fc or pBINPLUS-h-Fc plant expression vectors. (**a**) The schematic diagram of pBINPLUS-GA733-Fc and pBINPLUS-h-Fc vectors. p35S, cauliflower mosaic virus 35S promoter; UTR, 5′ UTR from the tobacco etch virus; SP, 30-aa plant ER signal peptide (MATQRRANPSSLHLITVFSLLAAVVSAEVD); KDEL, endoplasmic reticulum (ER) retention motif; t35S, cauliflower mosaic virus 35S terminator; NPT II, neomycin phosphotransferase II. (**b**) The transgenic plants were screened by PCR amplification of the expression cassettes. (**c**) Protein expression levels were determined by ELISA analysis with AP-conjugated goat anti-human IgG. Data represent mean + standard deviation (N = 3). (**d**) Western blot analysis with the GA733-2 specific antibody using leaves of the transgenic plants.

**Figure 2:**
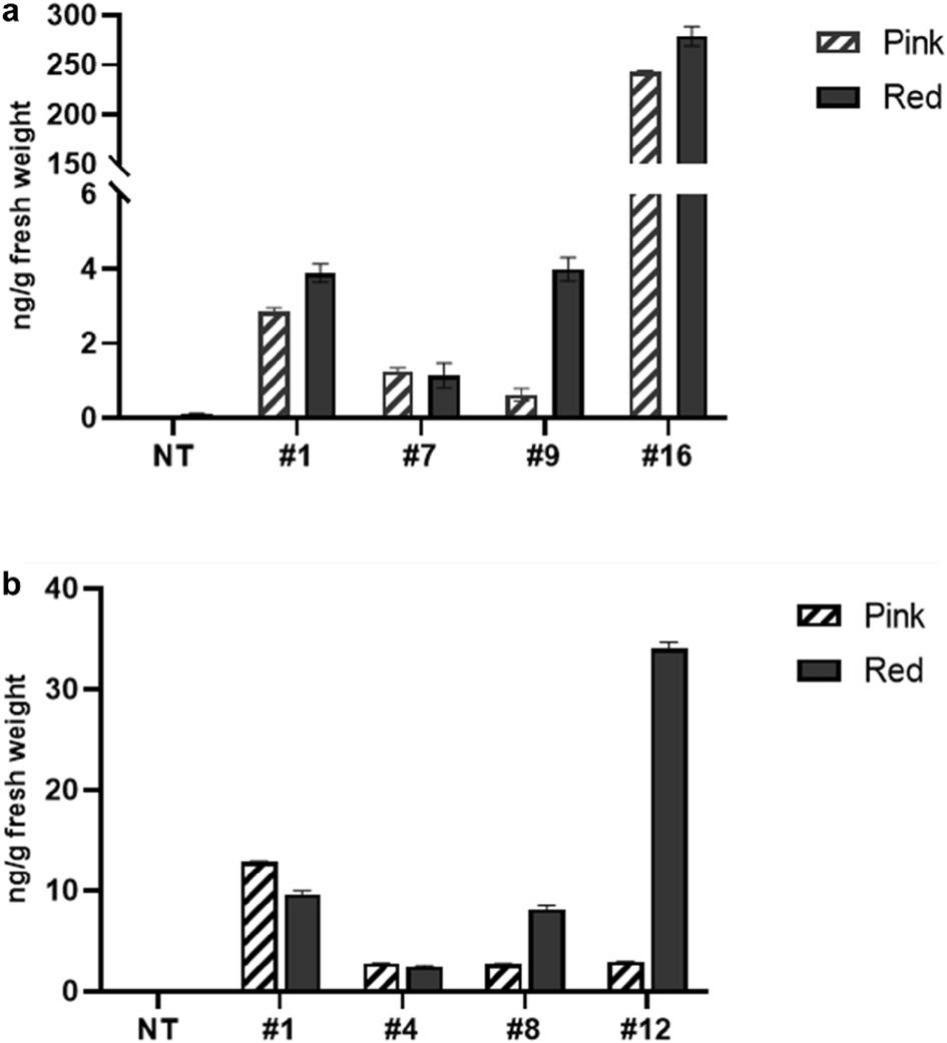
The rGA733-Fc and h-Fc protein expression levels in the transgenic tomato fruits. ELISA analysis was performed to quantify the protein expression level in the pink and red stage of transgenic fruits. (**a**) rGA733-Fc protein levels in fruits of *rGA733-Fc*^*OX*^ transgenic plants. (**b**) h-Fc protein levels in fruits of *h-Fc*^*OX*^ transgenic plants. Data represent mean + standard deviation (N = 3).

To generate transgenic tomato plants that stably express rGA733-Fc, a plant expression vector that expresses rGA733-Fc fused with ER-targeting signals and ER retention motif under the control of the 35S promoter (pBINPLUS-GA733-Fc)^41^ were transformed into *Solanum lycopersicum* cv. Micro-Toms plants using *Agrobacterium*-mediated genetic transformation (Fig. 1a). In addition, the pBINPLUS-Fc vector that expresses human-Fc (h-Fc) protein driven by *35S* promoter was also transformed into Micro-Toms plants (Fig. 1a). Integration of genetic cassette into tomato plants were validated by PCR analysis with specific primers for the expression cassettes. Through the process, we finally obtained total 16 transgenic plants containing rGA733-Fc expression cassette (*rGA733-Fc*^*OX*^) and 13 transgenic plants containing h-Fc expression cassette (*h-Fc*^*OX*^) (Fig. 1b and Fig. S1). Expression level of rGA733-Fc protein in the leaves of selected transgenic plants was determined by ELISA and western blot analysis (Fig. 1c,d, and Fig. S2). Among 16 *rGA733-Fc*^*OX*^ transgenic plants, *rGA733-Fc*^*OX*^ #16 showed the highest expression of rGA733-Fc (43.21 μg of rGA733-Fc/g fresh weight) (Fig. 1c and Table 1). In case of the *h-Fc*^*OX*^ transgenic plants, *h-Fc*^*OX*^ #1 produced 5.23 μg of h-Fc protein per gram of fresh weight (Fig. 1c and Table 1). Four independent transgenic plants that express corresponding recombinant proteins were chosen to monitor rGA733-Fc protein level in tomato fruits. Among the *rGA733-Fc*^*OX*^ transgenic plants, *rGA733-Fc*^*OX*^ #16 showed the highest expression of rGA733-Fc in both pink and red tomato fruits (Fig. 2a and Table 1). Similarly, *h-Fc*^*OX*^ #1 produced the highest level of h-Fc protein in both leaves and pink tomato fruits. However, *h-Fc*^*OX*^ #12 that produced relatively low h-Fc protein in leaves showed the highest expression level of h-Fc protein in red tomato fruits (Fig. 2b and Table 1).

**Table 1:** Quantification of the rGA733-Fc and h-Fc protein in transgenic tomato plants.

	*rGA733-Fc*^*OX*^				*h-Fc*^*OX*^			
	#1	#7	#9	#16	#1	#4	#8	#12
Leaves (µg/g FW)	10.02	1.97	4.76	43.21	5.23	3.33	3.98	0.66
Pink fruits (ng/g FW)	2.85	1.26	0.62	242.79	12.89	2.75	2.73	2.94
Red fruits (ng/g FW)	3.88	1.13	3.99	278.76	9.60	2.44	8.10	34.04

*rGA733-Fc*^*OX*^, rGA733-Fc overexpressing plants; *h-Fc*^*OX*^, ﻿human-Fc overexpressing transgenic plants.

### N-glycosylation pattern analysis of purified rGA733-Fc protein from transgenic tomato plants

**Figure 3:**
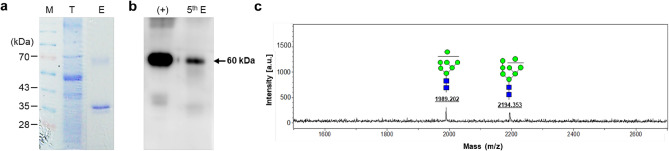
The rGA733-Fc protein purification and glycosylation pattern analysis. rGA733-Fc protein was purified from the transgenic tomato plants using a protein A column. (**a**) The eluted fractions were separated by SDS-PAGE and visualized by Coomassie brilliant blue staining. M, protein size marker; T, total soluble protein; E, purified rGA733-Fc protein. (**b**) rGA733-Fc protein in 5th eluted fraction was detected with the rGA733-2 antibody by western blot analysis. 5th E, 5th eluted fraction. (**c**) Glycosylation patterns of purified rGA733-Fc protein were analyzed by Matrix-assisted laser desorption/ionization time-of-flight (MALDI-TOF) mass spectrometry (MS). Blue square, *N*-acetylglucosamine (GlcNAc); green circle, mannose.

Purification of plant-derived protein is important for its application as vaccine candidate. Since rGA733-2 protein was fused with h-Fc domain, we used protein A column to purify rGA733-Fc proteins produced from transgenic tomato plants. Coomassie brilliant staining of the eluted fractions showed that most of the purified proteins were detected in 5th elution fraction (Fig. 3a and Fig. S3). The purified rGA733-Fc protein was confirmed by western blot analysis using a rGA733 specific antibody (Fig. 3b and Fig. S3). Final concentration of the purified rGA733-Fc was 8.69 μg/g from *rGA733-Fc*^*OX*^ #16 transgenic tomato plants. We next determine glycan structure of plant-derived rGA733-Fc protein through MALDI-TOF MS analysis. The observed masses (1989 and 2914 Da) were corresponded to Hex_7_HexNAc_2_ and Hex_8_HexNAc_2_, which were identified as oligomannose glycan structure. Thus, rGA733-Fc proteins obtained from *rGA733-Fc*^*OX*^ transgenic tomato plants contained oligomannose glycan structure, which are typically found from ER-associated proteins (Fig. 3c).

### Inhibitory effect of the plant-derived rGA733-Fc protein for colorectal cancer growth in tumor-bearing mice model

**Figure 4:**
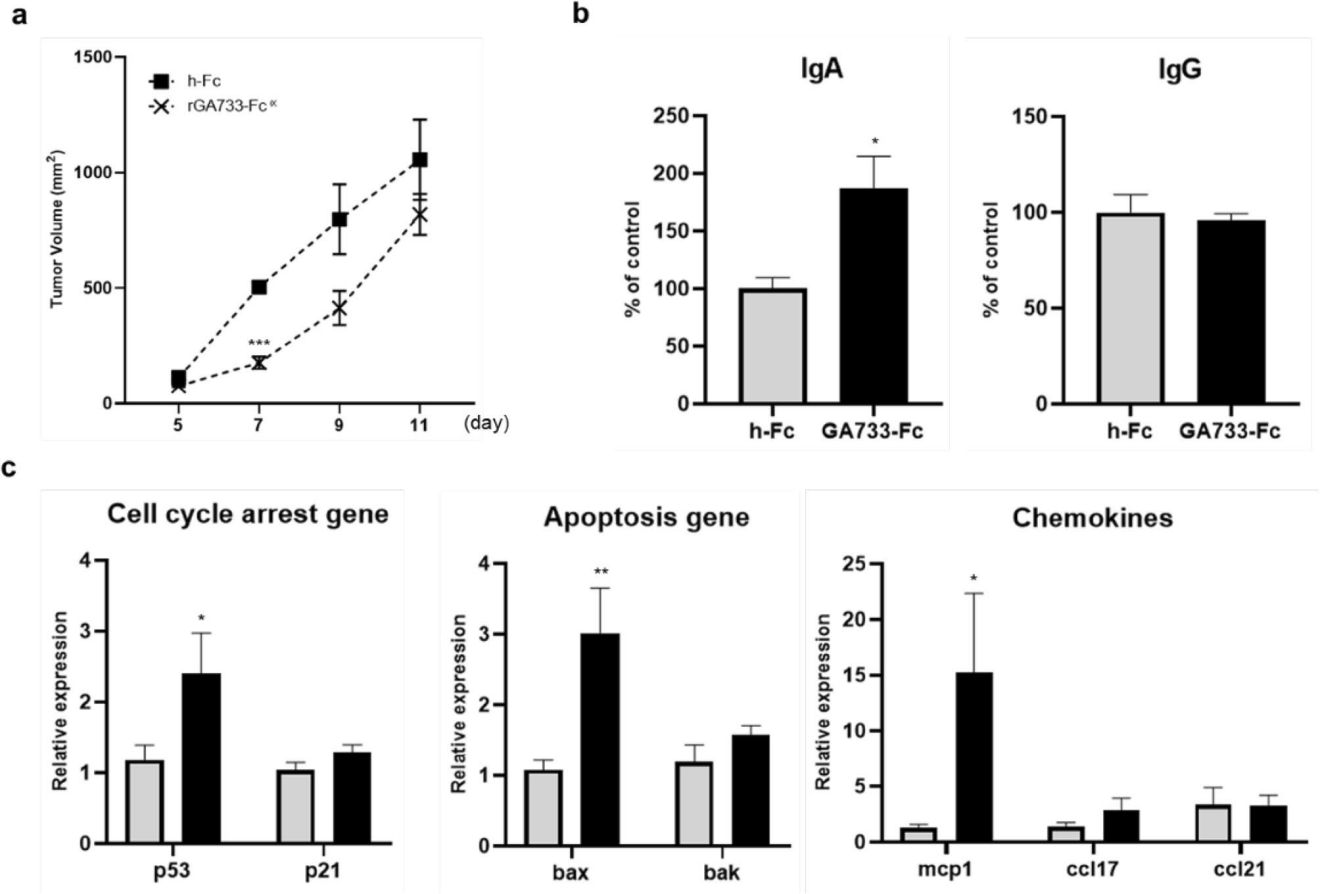
Effects of rGA733-Fc overexpression transgenic tomato fruits on colonic tumorigenesis in a colorectal cancer mouse model. rGA733-Fc overexpressing (*rGA733-Fc*^*OX*^ #16) transgenic tomato fruits were orally administered to C57BL/6J mice every other day for 4 weeks. The fruits of human-Fc overexpressing (*h-Fc*^*OX*^ #12) transgenic plants were used as a control for fruits of rGA733-Fc transgenic plants. The mice pre-treated with *rGA733-Fc*^*OX*^ or *h-Fc*^*OX*^ fruits were then injected subcutaneously with 1 × 10^6^ MC38 cells. Fecal extract and serum samples were obtained from mice at sacrifice at 14 days after MC38 cell injection. (**a**) The tumor volume was measured at the indicated time points by bilateral Vernier starting 14 days after MC38 cell injection. Data represent mean ± SEM (N = 3). (**b**) Levels of Immunoglobulin A (IgA) and IgG in fecal extracts and serum, respectively. IgA and IgG concentrations were measured by ELISA analysis. (**c**) Relative expression levels of genes involved in cell cycle arrest (p53 and p21), apoptosis (bax and bak), and chemokines (mcp1, ccl17, and ccl21). β-Actin was used as an internal control for normalization. Data represent mean ± SEM (N = 3). *p < 0.05, **p < 0.01, and ***p < 0.001.

To test effect of *rGA733-Fc*^*OX*^ transgenic plants for colorectal cancer development, ground red fruits of *rGA733-Fc*^*OX*^ transgenic plants were orally administered to C57BL/6 mice every other day for two weeks. Fruits of transgenic plants expressing h-Fc protein were used as a negative control. After two weeks of the treatments, MC38 colon cancer cells were injected subcutaneously (s.c.) in the right flank of pre-administrated mice. Colorectal tumor volumes were measured to determine whether the plant-derived rGA733-Fc inhibits colorectal cancer growth. The C57BL/6 mice pre-administered with fruits of *rGA733-Fc*^*OX*^ transgenic plants showed delayed tumor growth compared to the mice pre-treated with fruits of the *h-Fc*^*OX*^ transgenic plants (Fig. 4a).

To evaluate whether the plant-derived rGA733-Fc protein activates the immune responses in tumor-bearing mice model, a production of IgA and IgG was analyzed from the fecal extracts and serum samples, respectively. Pre-administration of *rGA733-Fc*^*OX*^ fruits significantly increased cellular IgA production compared to pre-administration of *h-Fc*^*OX*^ fruits (Fig. 4b). On the other hand, cellular IgG level was not significantly changed by *rGA733-Fc*^*OX*^ fruit treatments (Fig. 4b).

To understand the anti-cancer effect of the rGA733-Fc transgenic tomato fruits, we next examined expression level of the genes related to the cell cycle and apoptosis in the tumors of tumor-bearing mice (Fig. 4c). Expression of tumor suppressor gene p53 and p21 was increased in the tumor tissue of the mice pre-administered with plant-derived rGA733-Fc. Among two pro-apoptotic genes, expression of bax was significantly induced by oral administration of the plant-derived rGA733-Fc, while no significant change was detected on the expression of bak gene by the treatment. In addition, expression of chemokine mcp1 was significantly up-regulated by rGA733-Fc treatment. These data suggest that the plant-derived rGA733-Fc inhibited the growth of colorectal carcinoma potentially through activation of tumor suppressor and pro-apoptotic molecules (Fig. 4c).

## Discussion

Here, we proposed the potential application of the tomato expressing rGA733-Fc protein as an oral vaccine for prevention of colorectal carcinoma. To successfully produce GA733-2 colorectal antigen in tomato, we introduced pBINPLUS-GA733-Fc plant expression vector that expresses GA733-2 fused with a Fc domain and ER retention motifs (rGA733-Fc) under the control of 35S promoter into tomato plants. Four selected transgenic tomato plants accumulated rGA733-2 protein (Fig. 1 and Table 1). Among them, *rGA733-Fc*^*OX*^ #16 transgenic tomato plants accumulated significantly higher rGA733-Fc protein in leaves, and the rGA733-Fc protein level in leaves was even higher than that in transgenic tobacco plants transformed with same vector^41^. Moreover, the *rGA733-Fc*^*OX*^ #16 transgenic tomato plants accumulated fivefold more rGA733-Fc protein in fruits. The level of rGA733-Fc in fruits was maintained at a similar level at the end of maturity (Fig. 2 and Table 1). This finding suggests that a more powerful edible vaccine can be developed to treat colorectal carcinoma using tomato. The rGA733-Fc protein level was significantly high in leaves compared to fruits in the *rGA733-Fc*^*OX*^ transgenic plants (Table 1). Even though the 35S promoter has been regarded as a constitutive promoter, its activity decreased during fruit ripening in tomato plants^42^. The use of a promoter showing higher activity during fruit ripening will be helpful to further improve rGA733-Fc protein production in tomato fruits.

For successful development of plant-derived vaccine, it is crucial to reduce the potential for safety issue. Plant specific glycosylation pattern, such as β-1,2-xylose and α-1,3-fucose, often act as allergen for mammalian system^43,44^. The N-glycosylation patterns are known to be incorporated into plant protein in Golgi^43^. Thus, directing recombinant protein into ER might be effective to avoid unnecessary complex glycosylation on recombinant protein^45^. Moreover, oligomannose glycan patterns processed in ER improve antigen presentation and enhance the immune response through perception by mannose receptor on macrophages and dendritic cells^46^. Ishii and Kojima reported that oligomannose-coated liposomes showed enhanced immunization effects compared to uncoated liposomes^47^. Thus, oligomannose glycan patterns detected from rGA733-Fc would be also helpful for effective delivery of orally treated rGA733-Fc to immune system. It has been reported that fusion with KDEL ER retention motif is sufficient to target rGA733-Fc into the ER^40^. Similarly, rGA733-Fc fused with KDEL ER retention motif possessed oligomannose glycan structure (Fig. 3c), which is a typical glycosylation pattern detected in ER-localized protein. Two different glycosylation pattern of rGA733-Fc produced in tomato plants were also observed in rGA833-Fc produced in tobacco plants^41^, suggesting that translational fusion of recombinant protein with ER-retention motif is sufficient to avoid unnecessary complex glycosylation occurred in plants.

Fc fragment fusion has several advantage for expression of recombinant protein, such as improvement of stability and yield of recombinant protein^48,49^. In addition, Fc fragment fusion can be used for one step purification of recombinant protein with protein A column^48^. SDS-PAGE analysis confirmed that rGA733-Fc protein was successfully purified with protein A column (Fig. 3a). In addition to rGA733-Fc protein, additional protein band was also detected from protein A column purified sample. It has been reported that Fc fusion with recombinant protein results in production of degradation products^40,49^. ER targeting of recombinant protein fused with Fc fragment generally reduces production of degradation products^40,50^. For example, ER targeting of rGA733-Fc protein greatly reduced the formation of degradation products in tobacco plants^40,41^. Different with tobacco system, ER targeting of rGA733-Fc still produced significant amount of degradation products in tomato system (Fig. 3a). Additional process, such as size exclusion chromatography, during purification for excluding the degraded products will be helpful for large scale production of rGA733-Fc protein.

To confirm the anti-cancer effects of tomato fruits producing rGA733-Fc protein, colorectal cancer was induced in the rGA733-Fc-immunized C57BL/6J mice. Pre-administration of the *rGA733-Fc*^*OX*^ transgenic tomato fruits (0.1 g per mice) significantly delayed development of colon cancer in the colorectal cancer mouse model (Fig. 4a). Similar with oral administration of rGA733-Fc through transgenic tomato fruits, injection of purified GA733-Fc produced in either mammalian and plant system successfully induced immune responses in mice^40^. The sera of mice immunized with plant-derived GA733-Fc showed similar immunogenicity compare with the sera of mice immunized with mammalian-derived GA733-Fc. In addition, the sera of mice immunized either insect cell-derived or plant-derived GA733-Fc showed similar binding affinity to colorectal cancer cell^51^. These results indicate that plant-derived GA733-Fc is as efficient as mammalian or insect cell-derived GA733-Fc for inducing immune response in mice.

The pre-administration of *rGA733-Fc*^*OX*^ transgenic tomato fruits also activated immune responses (Fig. 4b) and induced expression of cell cycle arrest and pro-apoptotic genes in the colorectal cancer-induced mice (Fig. 4c). Generally, IgA is mainly produced and secreted by B cells in mucosal area including intestine and respiratory tracts, and it is well known to play important roles in mucosal immune responses^52^. On the other hand, IgG is involved in immune responses of blood and body tissue^53^. In this study, we showed that the production of IgA was increased by the oral-administration of *rGA733-Fc*^*OX*^ fruits in the mucosal area, while no difference was observed in the IgG production of blood serum. Thus, these data indicate that the oral-administration of *rGA733-Fc*^*OX*^ fruits lead to enhance the immune responses in the mucosal area, not in the whole-body area. Recent studies report that not only IgG but also IgA have therapeutic potentials in antibody-based cancer immunotherapy using tumor antigen-targeting monoclonal antibodies^53,54^. The antigen-targeting monoclonal antibodies enhance cancer cytotoxicity through inducing complement activation, antibody-dependent cellular cytotoxicity and antibody-dependent cellular phagocytosis^55^. Although, further studies are necessary to clarify the direct mechanisms of IgA antibody-mediated anti-cancer effect, we suggest that the oral-administration of rGA733-Fc increases the production of tumor antigen-targeting antibodies and may have a therapeutic potential for colon cancer patients. Collectively, we suggest that tomato fruits producing rGA733-Fc protein have a potential to be used as an edible vaccine to suppress the development of colorectal cancer. In addition, *rGA733-Fc*^*OX*^ transgenic tomato may function as a research tool that will help to further elucidate the anti-cancer mechanism of rGA733-Fc as an oral vaccine against various types of cancer.

## Methods

### Plant expression vector

A truncated form of human colorectal carcinoma antigen GA733-2 (aa 17–266)^41,56,57^ and Fc fragment of human IgG1 (Val97-Gly328, GenBank accession No.AY172957) were used in this study. The GA733-2 fused with human Fc-and ER signal peptide (rGA733-Fc) were amplified using the primers GA733-Fsal (5′-ACGTCGACATGGATACGGCG-3′), Ig-Fsal (5′-ACGTCGACGTTGAGCCCAAATCTTG-3′) and KDEL-Rsnab (5′-TGTACGTATCAGAGTTCATCTTT-3′). The PCR products were introduced into a plant expression vector pBINPLUS through SalI and SacI restriction enzyme sites. The final constructs named as pBINPLUS-GA733-Fc and pBINPLUS-h-Fc^41^ were used for tomato transformation.

### Agrobacterium-mediated transformation of *Solanum lycopersicum* cv. Micro-Tom

Wild tomato seeds (*Solanum lycopersicum* cv. Micro-Tom) were provided by Sanghyeob Lee of Sejong University in Republic of Korea (ROK). WT tomato plants and transgenic lines were grown under controlled growth room condition with 16 h light/8 h dark cycle (22–24 °C). Collection of plant material, must comply with relevant institutional, national, and international guidelines and legislation. This experiment was conducted with permission at Chonnam National University (35° 10′ N, 126° 53′ E) in Gwangju, Republic of Korea. The plant materials and residues were discarded according to the biosafety guidelines. Transgenic tomato plants expressing rGA733-Fc or h-Fc were produced using the *Agrobacterium*-mediated transformation method^58^. Cotyledons from a sterile cultured three-week-old tomato plants were cut into two pieces and inoculated with *Agrobacterium tumefaciens* bacteria solution. After the inoculation, the explants were placed on co-culture medium [3% sucrose (Duchefa, RV Haarlem, Netherlands), 4.4 g/L MS salt (Duchefa, RV Haarlem, Netherlands), 0.1 mg/L IAA (Duchefa, RV Haarlem, Netherlands), 1 mg/L zeatin (Duchefa, RV Haarlem, Netherlands), and 200 μM acetosyringone (MBcell, Seoul, Korea), pH 5.2]. After 2 days of co-cultivation, the cotyledon pieces were transferred to shoot induction medium [3% sucrose, 4.4 g/L MS salt, 50 mg/L kanamycin (Biosesang, Sungnam, Korea), 0.1 mg/L IAA, 1 mg/L zeatin, and 250 mg/L cefotaxime (Biosesang, Sungnam, Korea), pH 6.0], and the explants were transferred to fresh shoot induction medium every 2–3 weeks until regenerated shoot appeared. The regenerated shoots were then transferred into shoot elongation medium (1.5% sucrose, 4.4 g/L MS salt, 50 mg/L kanamycin, and 250 mg/L cefotaxime, pH 6.0) for further development. The callus with well-developed shoots were then transferred into root induction medium [1.5% sucrose, 4.4 g/L MS salt, 50 mg/L kanamycin, 2 mg/L IBA (Duchefa, RV Haarlem, Netherlands), and 50 mg/L cefotaxime, pH 6.0]; the regenerated plantlets with roots were transferred to soil and grown in a growth room (16 h light/8 h dark cycle, 22–24 °C).

### Genomic DNA extraction and PCR analysis of transgenic plants

Genomic DNA was isolated from 300 mg transgenic tomato leaves to confirm the insertion of the transgene cassette. Transgenic tomato leaves were ground to fine powder with liquid nitrogen, and mixed well with 700 µl of DNA extraction buffer [0.05 M Tris–HCl, pH 7.6 (Biosesang, Sungnam, Korea), 0.5% SDS (Sigma-Aldrich, St. Louis, MO, USA), 0.1 M NaCl (Sigma-Aldrich, St. Louis, MO, USA), 0.05 M EDTA (Sigma-Aldrich, St. Louis, MO, USA), and 0.1 M β-mercaptoethanol (Sigma-Aldrich, St. Louis, MO, USA)]. The mixtures were incubated at 50 °C for 10 min with inverting every 2 min. The extracts were thoroughly mixed with 500 µl of phenol:chloroform:isoamylalcohol (25:24:1, Biosesang, Sungnam, Korea). The samples were centrifuged at 13,000 rpm for 15 min, and then upper aqueous phase was transferred to fresh micro centrifuge tubes. 2 volumes of 100% ethanol were added to the tubes and the samples were mixed by gently inversion. The tubes were centrifuged at 13,000 rpm for 10 min at 4 °C. The pellet was dissolved in distilled water after washing with 75% ethanol. The presence of the rGA733-Fc or human Fc expression cassette in plants was determined by PCR analysis using 35S-F (5′-GGATGACGCACAATCCCACTATCC-3′), Fc-R (5′-AGGGAGAGGCTCTTCTGCGT-3′) primers. PCR reactions were carried out by following conditions; preheating at 95 °C for 5 min, 34 amplification cycles (95 °C for 30 s, 55 °C for 30 s, and 72 °C for 1 min), final incubation at 72 °C for 10 min.

### rGA733-Fc protein purification

The transgenic plants harboring rGA733-Fc expression cassette were ground in 0.5 × PBS buffer (8.17 mM Na_2_HPO_4_, 1.34 mM KCl, 0.47 mM K_3_PO_4_, 68.45 mM NaCl, pH 7.4). The soluble fraction was dialyzed in binding buffer (10 mM NaH2PO4, 150 mM NaCl, pH 8.2). After dialyzation, total soluble proteins were purified via Affi-Gel^®^ Protein A Gel column (Bio-Rad Laboratories, Hercules, CA, USA) at a flow rate of 0.5 ml/min. The glycosylation patterns of purified rGA733-Fc proteins were analyzed by Matrix-assisted laser desorption/ionization time-of-flight (MALDI-TOF) mass spectrometric (MS) analysis.

### Western blot analysis and quantification of rGA733-Fc and human Fc proteins in transgenic plants

Total soluble proteins were isolated from 200 mg of tomato leaves. The harvested tomato leaves were homogenized using a TissueLyser II (Qiagen, CA, USA) with one volume of Bradly buffer^59^ at 30 Hz for 5 min. 50 μg of total soluble proteins were separated on a 10% or 12% SDS polyacrylamide gel, and transferred to a polyvinylidene fluoride membrane (PVDF; GE Healthcare, USA). The PVDF membrane was incubated with a blocking solution [1% bovine serum albumin (BSA) and 5% skim milk] at 25 °C for 3 h and then washed with TBS-T (150 mM NaCl, 2.5 mM KCl, 25 mM Tris base, and 0.1% Tween-20, pH 7.4). The rabbit rGA733-2 specific antibody^57^ was used as a primary antibody (1:5000 dilution) and the HRP-conjugated goat anti-human IgG was used as a secondary antibody (1:5000 dilution, Thermo Scientific, Waltham, USA). The rGA733-Fc proteins were visualized using an Immobilon western chemiluminescent HRP system (Millipore, Billerica, MA, USA) and a LAS-3000 luminescent image analyzer (Fujifilm, Tokyo, Japan).

Quantification of rGA733-Fc protein was performed as described previously^41^. 10 µg/ml of total soluble proteins from transgenic tomato leaves and fruits were plated on a 96-well immunoassay plate. The individual wells were blocked with 200 µl of 5% skim milk in PBS buffer (16.34 mM Na2HPO4, 2.68 mM KCl, 0.94 mM K3PO4, 136.9 mM NaCl, pH 7.4) for 2 h. The immunoplate was then incubated with 1:2000-diluted rabbit rGA733-2 specific antibody, and then incubated with 1:2000-diluted AP-conjugated goat anti-human IgG (Thermo Scientific, Waltham, USA). The concentration of immunoblotted proteins was determined with p-nitrophenyl phosphate (1 mg/ml; Sigma-Aldrich, St. Louis, MO, USA) as a substrate by measuring an absorbance at 450 nm using a VERSA max microplate reader (Molecular Devices, California, USA).

### N-glycan analysis

Samples were digested into glycopeptides by pepsin, followed by peptide N-glycosidase (PNGase) A (Roche, Basel, Switzerland) treatment, as previously described^60^. The released N-glycans were purified using graphitized carbon resin from Carbograph (Alltech, Lexington, MA). Purified glycans were dried and then re-dissolved in a mixture of 90 μl dimethyl sulfoxide (DMSO), 2.7 μl water, and 35 μl iodomethane for solid-phase permethylation using a spin-column method^61^. After the process previously described, the chloroform layer containing permethylated glycans was dried and resuspended in 4 μl of a 50% methanol solution. Then, it was mixed in equal volume with the matrix 2,5-dihydroxybenzoic acid prepared in 1 mM sodium acetate solution for matrix-assisted laser desorption ionization-time of flight (MALDI-TOF) mass spectrometry. The resulting mixtures were applied onto a MALDI MSP 96 polished steel Chip (Bruker Daltonik GmbH, Bremen, Germany) and dried. The mass spectrometry was performed in the reflector positive ion mode using a Microflex (Bruker Daltonik). All mass spectra were acquired at a 20 kV accelerating voltage using the method recommended by the manufacturer. The glycan compositions were identified from the observed masses by using the glycan molecular weight search program on the website of Consortium for Functional Glycomics (http://www.functionalglycomics.org/). The most possible glycan structures were provided, based on the mass information for plant N-glycans^62^.

### Mouse immunization and induction of colorectal cancer

Potential anti-cancer effects of rGA733-Fc was tested in a colorectal cancer mouse model with transgenic tomato fruits. Seven-week-old male C57BL/6 mice were purchased from the Damool Animal Breeding Company (Daejeon, Korea). Three C57BL/6 mice per each condition were immunized every other day for 4 weeks with 100 mg of transgenic tomato fruit extract (*rGA733-Fc*^*OX*^ #16 and *h-Fc*^*OX*^ #12) in 100 μl PBS by oral injection. Fourteen days later, the mice were hypodermically injected with 1 × 10^6^ of MC38 cells. Beginning from 5 days after MC38 cell injection, tumor volume was measured for 13 days. At the end of the experiment (D28), the mice were euthanized. Total IgA and IgG were extracted from fecal and serum, respectively, and quantified by ELISA. The experiment using mice was carried out to the guidelines of the Institutional Animal Care Committee of Chonnam National University and approved by the Ethics Committee of Chonnam National University (CNU IACUC-YB-2013-07). The study was carried out in compliance with the ARRIVE guidelines.

### Real-time PCR analysis

Total RNAs were extracted from cancer tissues using TRIzol reagent (MRC, Cincinnati, OH, USA), according to the manufacturer's instruction. 1 μg of total RNAs was used for cDNA synthesis using the QuantiTect^®^ Reverse Transcription Kit (Qiagen, CA, USA) and quantitative real-time PCR (qRT-PCR) analysis was performed with the QuantiTect^®^ SYBR^®^ Green PCR Kit (Qiagen, CA, USA) and the Rotor-Gene 6000 real-time amplification operator (Qiagen, CA, USA). The primers used in this study were purchased from Cosmo Genetech (Seoul, Korea) and the sequence of the primers is as follows: β-actin (forward, 5′-GACGGCCAGGTCATCACTAT-3′ and reverse, 5′-AGTCCGCCTAGAAGCACTTG-3′), p53 (forward, 5′-CATCACCTCACTGCATGGAC-3′ and reverse, 5′-CTCGGGTGGCTCATAAGGTA-3′), p21 (forward, 5′-TCCAGACATTCAGAGCCACA-3′ and reverse, 5′-GCTCAGACACCAGAGTGCAA-3′), bax (forward, 5′-ACCAGCTCTGAACAGATCATG-3′ and reverse, 5′-ACTTTAGTGCACAGGGCCTTG-3′), bak (forward, 5′-ACGAACTCTTCACCAAGATCGCCT-3′ and reverse, 5′-TCAAACCACGCTGGTAGACGTACA-3′) mcp1 (forward, 5′-CACCTGCTGCTACTCATTCA-3′ and reverse, 5′-CACTGTCACACTGGTCACTCCTAC-3′), ccl17 (forward, 5′-CAGGAAGTTGGTGAGCTGGTATA-3′ and reverse, 5′-TTGTGTTCGCCTGTAGTGCATA-3′), ccl21 (forward, 5′-AGACTCAGGAGCCCAAAGCA-3′ and reverse, 5′-GTTGAAGCAGGGCAAGGGT-3′). The β-actin was used as internal control for normalization and relative expression was calculated by ΔΔCt method.

### Statistical analysis

Experimental results were presented as mean ± standard error of the mean (SEM) and analyzed with unpaired Student’s *t* test by GraphPad Prism (Version 8.4.2, GraphPad Prism Software Inc., La Jolla, CA, USA). The data were considered to be statistical significance *p < 0.05, **p < 0.01, and ***p < 0.001.

## Supplementary Information

Supplementary Figures.

## Data Availability

The data presented in this study are available on request from the corresponding author. The data are not publicly available due to privacy.
