# Supplementary material for: Immunotherapeutic effects of recombinant colorectal cancer antigen produced in tomato fruits
Source: Sci Rep. 2022 Jun 13;12:9723. doi: 10.1038/s41598-022-13839-1 (PMC9192744; doi:10.1038/s41598-022-13839-1)
Supplement: Supplementary file 1 — Supplementary Figures. [file 41598_2022_13839_MOESM1_ESM.pdf]

Immunotherapeutic effects of recombinant colorectal cancer antigen produced in tomato fruits

Se Hee Park[1,†], Kon-Young Ji[2,†], Seo Young Park[1], Hyun Min Kim[1], Sang Hoon Ma[1], Ju Hui Do[1], Hyuno Kang[3], Hyung Sik Kang[1], Doo-Byoung Oh[4,5], Jae Sung Shim[1*], Young Hee Joung[1*]

[1]School of Biological Sciences and Technology, Chonnam National University, Gwangju 61186, Korea

[2]Herbal Medicine Research Division, Korea Institute of Oriental Medicine, Daejeon 34054, Korea

[3]Division of Analytical Science, Korea Basic Science Institute (KBSI), Daejeon 34133, Republic of Korea

[4]Environmental Disease Research Center, Korea Research Institute of Bioscience and Biotechnology (KRIBB), Daejeon 34141, Korea

[5]Department of Biosystems and Bioengineering, KRIBB School of Biotechnology, University of Science and Technology (UST), Daejeon 34113, Korea

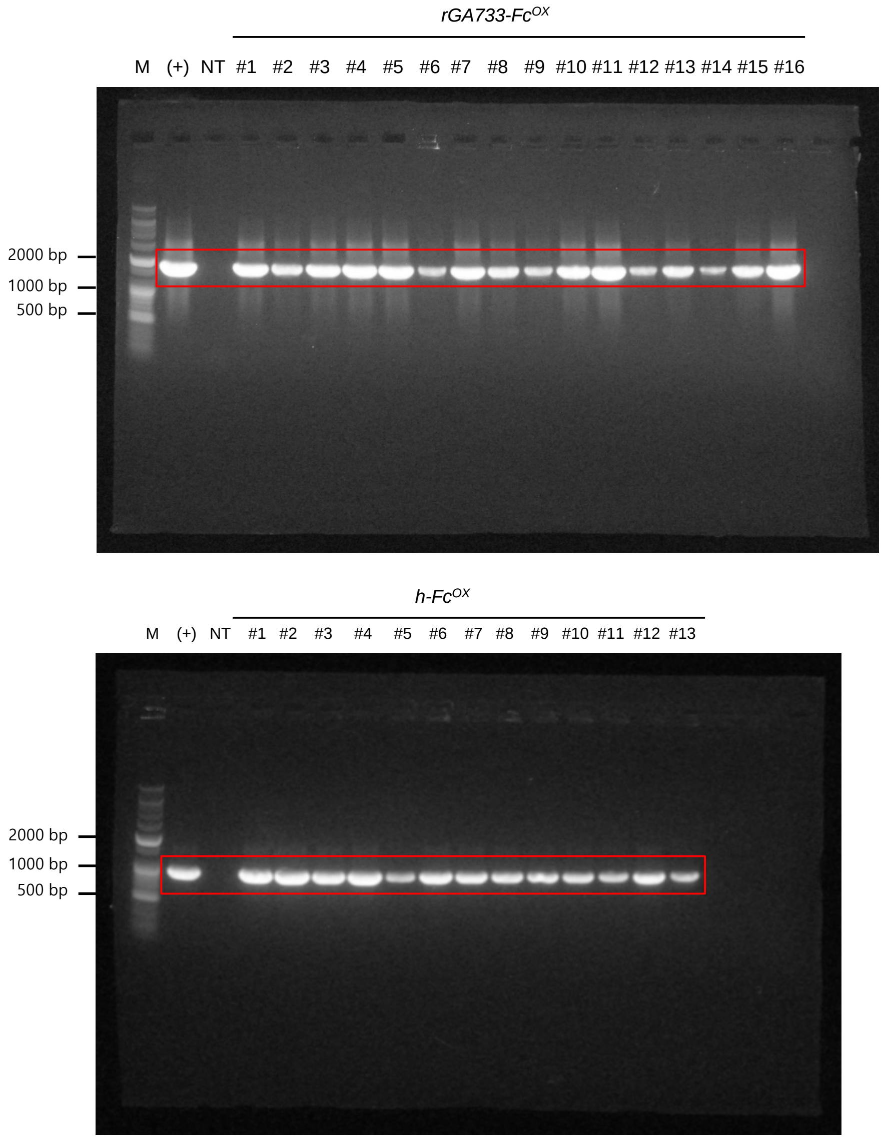

Supplementary Figure S1. Screening of transgenic tomato plants. Full size image of gels for Figure 1b was presented. The parts of the gel used in the manuscript are indicated by red boxes. Size of the DNA ladder was represented on left or right of the each figures. *rGA733-Fc*^{OX}, transgenic plants expressing *rGA733-Fc*; *h-Fc*^{OX}, transgenic plants expression *h-FC*; M, Marker; +, positive control; NT, negative control.

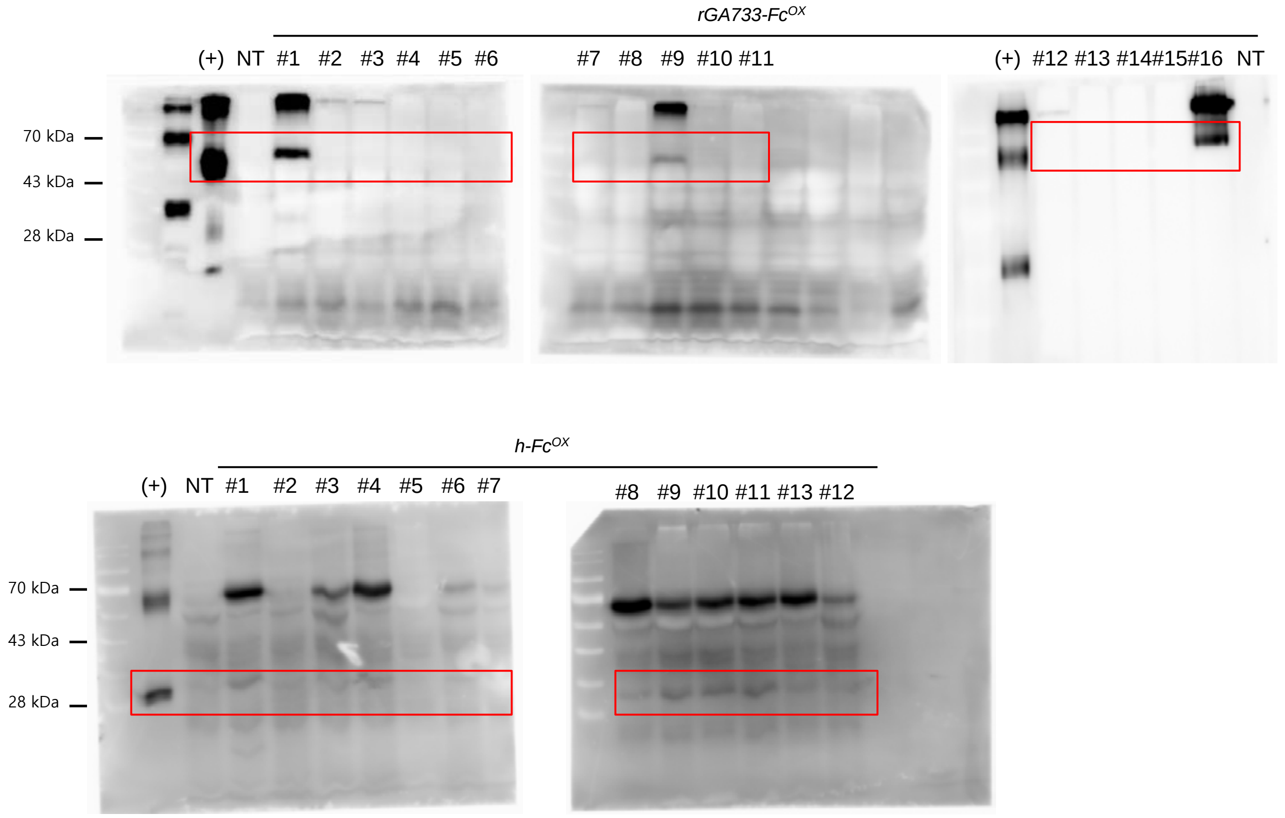

Supplementary Figure S2. Expression of rGA733-Fc and h-Fc in transgenic plants. Full size image of western blots for Figure 1d was presented. The parts of the blot used in the manuscript are indicated by red boxes. Size of the protein marker was represented on left of the figures. *rGA733-Fc*^{OX}, transgenic plants expressing *rGA733-Fc*; *h-Fc*^{OX}, transgenic plants expression *h-FC*; +, positive control; NT, negative control.

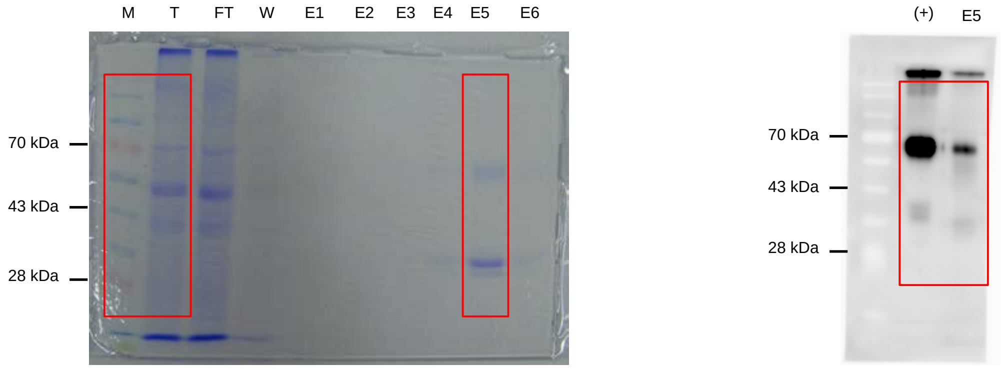

Supplementary Figure S3. Purification of rGA733-Fc. Full size image western blot for Figure 3a and b was presented. The parts of the blot used in the manuscript are indicated by red boxes. Size of the protein marker was represented on left of the figures. M, size marker; T, total soluble fraction; FT, flow through; W, flow-through by washing; E1-E6, eluted fractions. +, positive control, E5, 5th eluted fraction.
